# Dickkopf 4 Alone and in Combination with Leucyl-tRNA Synthetase as a Good Prognostic Biomarker for Human Colorectal Cancer

**DOI:** 10.1155/2023/9057735

**Published:** 2023-04-15

**Authors:** Su-Jeong Park, Jun Gi Cho, Sang-Heum Han, Yu-Mi Kim, Min-Gyoung Pak, Mee-Sook Roh, Joo-In Park

**Affiliations:** ^1^Department of Biochemistry, Dong-A University College of Medicine, 32, Daesingongwon-ro, Seo-gu, Busan 49201, Republic of Korea; ^2^Department of Translational Biomedical Sciences, Graduate School, Dong-A University, 32, Daesingongwon-ro, Seo-gu, Busan 49201, Republic of Korea; ^3^Peripheral Neuropathy Center, Dong-A University, 32, Daesingongwon-ro, Seo-gu, Busan 49201, Republic of Korea; ^4^Department of Preventive Medicine, Dong-A University College of Medicine, 32, Daesingongwon-ro, Seo-gu, Busan 49201, Republic of Korea; ^5^Department of Pathology, Dong-A University College of Medicine, 32, Daesingongwon-ro, Seo-gu, Busan 49201, Republic of Korea

## Abstract

The prognosis of patients with colorectal cancer (CRC) is affected by invasion and metastasis. Leucyl-tRNA synthetase (LARS) was shown to be related to the growth and migration of lung cancer cells. Dickkopf 4 (DKK4) is known as a Wnt/*β*-catenin pathway inhibitor, and its upregulation was reported in several cancers. However, the clinical significance of LARS and DKK4 in human CRC has not been clearly defined. We investigated the expression of LARS and DKK4 by immunohistochemical staining in tissue microarrays from 642 primary CRC patients and analyzed the relationship between their expression and the clinicopathological characteristics of CRC patients. LARS and DKK4 expressions were not related to gender, age at surgery, histologic grade, size, tumor location, tumor invasion, or metastasis, but LARS expression was significantly correlated with TNM stage, N stage, and lymph node metastasis. DKK4 expression was inversely related to the TNM stage and N stage. Survival analysis demonstrated that the OS and DFS in the LARS high expression group were not different compared to the LARS low expression group. OS and DFS in the DKK4 high expression group were significantly higher than in the DKK4 low expression group. In addition, OS and DFS in the group with the combination of the LARS high/DKK4 low expression were significantly lower than in the LARS high/DKK4 high expression group. The low expression of DKK4 alone can be used as a predictor of relapse in CRC patients. In addition, DKK4 low expression in the case of LARS high expression can be used as a poor prognostic factor in CRC patients. Thus, our findings suggest that DKK4 alone or in combination with LARS at diagnosis may be a useful prognostic factor for CRC.

## 1. Introduction

Colorectal cancer (CRC) is the fourth most common cancer in the world [1]. The prognosis of CRC is dependent upon invasion, lymph node (LN) involvement, and distant organ metastasis. The sequence of CRC progression and some of the involved mechanisms were revealed by recent molecular studies [2, 3]. However, molecular biomarkers predicting relapse, regional invasion, and metastasis in CRC are not well known. Thus, many researchers have focused on identifying novel molecular biomarkers for more aggressive CRC phenotypes.

Leucyl-tRNA synthetase (LARS), which contributes to protein synthesis by catalyzing the ligation of leucine to its corresponding tRNA, senses intracellular leucine and activates mechanistic target of rapamycin complex 1 (mTORC1) through direct binding to RagD GTPase, an important mediator of the amino acid-dependent mTORC1 pathway [4, 5]. LARS was reported to be closely related to the growth and migration of lung cancer cells by observing the reduced migration and colony formation from LARS1 siRNA knockdown in a lung cancer cell line [6]. LARS expression has no reported biological or clinical implications in CRC patients, even though a few compounds targeting LARS as potential anticancer agents have been developed and their action mechanisms are studied [7–13].

The Dickkopf (DKK) gene family encodes secreted proteins in vertebrates (DKK1 to DKK4) [14, 15]. The Wnt (wingless-type mouse mammary tumor virus integration site family) signaling plays a role in various processes including embryonic development and the regulation of homeostasis and carcinogenesis [16]. *β*-Catenin binds to T-cell factor/lymphoid enhancer factor (TCF/LEF), resulting in the transcriptional activation of target genes involved in the Wnt signaling pathway [17, 18]. DKK protein family members (DKK1, DKK2, and DKK4) are known to inhibit Wnt/*β*-catenin through binding to lipoprotein receptor-related protein 5/6 (LRP 5/6) [19, 20]. Increasing evidence has demonstrated that DKK1 or DKK3 is involved in the carcinogenesis of various organs including head and neck squamous cell carcinoma and esophageal cancers [21–23]. Especially, it was reported that DKK1 protein expression was correlated with the poor overall survival (OS) of patients with esophageal squamous cell carcinoma [23]. Wang et al. demonstrated that DKK4 was overexpressed in epithelial ovarian cancer and promoted invasion through the activation of JNK [24]. A previous study showed that DKK4 expression was increased in CRC using clinical samples from a small number of patients and that the activation of the Wnt/*β*-catenin pathway induced DKK4 expression *in vitro*, suggesting that DKK4 expression may reflect an activated Wnt/*β*-catenin pathway in CRC [25]. It was also shown that DKK4 increased cell migration and invasion [26, 27]. Further studies demonstrated that DKK4 expression may contribute to chemotherapy resistance in CRC [28, 29]. A recent report showed that DKK4 expression was associated with differentiation and LN metastasis in CRC [30]. Although increasing evidence has revealed that DKK4 promotes cancer progression and the acquisition of resistance to chemotherapy, some reports showed that DKK4 inhibited cell proliferation, migration, and invasion in cancer [31, 32].

The link between DKK4 expression and the clinical characteristics of CRC patients was first indicated by the correlation between LN metastasis and the increased expression of DKK4 [30]. However, the molecular mechanisms by which DKK4 affects cell proliferation or invasion remain unclear, and the clinical significance of DKK4 in CRC has not been well established.

In our present study, we investigated LARS and DKK4 expressions in 642 primary CRC tissue microarrays. We further examined the association between their expression and clinicopathological factors including OS and disease-free survival (DFS) in CRC patients.

## 2. Materials and Methods

### 2.1. Patients and Tissue Samples

Six hundred forty-two consecutive eligible CRC patients who underwent surgery at Dong-A University Hospital in 2002–2011 were enrolled in this study. The eligible patients had no family history of CRC and had not received radiotherapy or preoperative chemotherapy. Patients with inflammatory bowel disease or familial adenomatous polyposis and synchronous colorectal or extracolorectal cancer and those lost to follow-up were excluded. Tissue samples from CRC patients were formalin-fixed and paraffin-embedded. Information about age, sex, histologic grade, size, location, tumor-node-metastasis (TNM) stage [33], N stage, relapse, and survival was retrieved by reviewing the medical reports. This study was performed in accordance with the Declaration of Helsinki and was approved by the Institutional Review Board (IRB) of Dong-A University (approval number BR-001-02).

### 2.2. Tissue Microarrays and Immunohistochemistry

Tissue microarrays were prepared as previously described [34, 35]. Sections (4 *μ*m thick) were subjected to immunohistochemical analysis for LARS and DKK4 using the avidin-biotin-peroxidase complex method [35]. All sections were deparaffinized, rehydrated, and antigen-retrieved as previously described [35]. Endogenous peroxidase activity was blocked with 5% hydrogen peroxidase for 10 min, and then, the samples were incubated with a primary antibody for 1 hour at room temperature (RT). The primary antibodies were a rabbit polyclonal antibody against LARS (diluted 1 : 200; Proteintech Group, Inc. IL, USA) and a rabbit polyclonal antibody against DKK4 (diluted 1 : 400; Abcam, UK). An Envision™Chem™ Detection Kit (DakoCytomation, CA, USA) was used for the secondary antibody at RT for 30 min. After washing the tissue samples in Tris-buffered saline for 10 min, 3,3'.′-diaminobenzidine was used as a chromogen, followed by the application of Mayer's hematoxylin as a counterstain. Positive controls for LARS and DKK4 were colon cancer and normal kidney, respectively. The negative control was obtained by using a buffer instead of a primary antibody.

### 2.3. Immunohistochemical Interpretation

The percentage and intensity of immunoreactive cancer cells in each core were recorded, and the final value of the positive cancer cells was determined as the mean of the immunoreactivity of three cores as described as previously [35]. All slides were independently evaluated by two experienced pathologists who were blinded to the clinicopathological findings. There were only minor discrepancies in the evaluations, which were resolved by reevaluation under a multihead microscope until achieving a consensus. The percentage of positive cancer cells and staining intensity were assessed. The intensity of staining was scored visually and stratified as follows: negative, weak, moderate, or strong (if it was obviously positive at 20x magnification). The immunoreactivity of LARS and DKK4 was defined as cells showing cytoplasmic staining in the cancer tissue with minimal background staining. Tumors with moderate or strong intensity in >10% of the tumor cells were recorded as having high immunoreactivity for LARS or DKK4 because their immunoreactivity was evenly distributed within a tumor but varied in intensity.

### 2.4. Statistical Analysis

The chi-squared test was used to analyze the relationship between the clinical characteristics and the immunohistochemistry data. The samples were divided into two groups based on high or low LARS and DKK4 staining. We performed between-group comparisons of the numbers of samples, clinicopathological characteristics, OS, and DFS. OS was defined as the length of time from surgery to death or last follow-up and DFS as the length of time from surgery to initial disease recurrence. Survival analysis was performed using the Kaplan-Meier method, and statistical significance was evaluated by the log-rank test. We used the Cox proportional hazard model to perform multivariate analysis including covariates that showed statistical significance in univariate analysis. A *p* value of <0.05 was considered to indicate statistical significance in all analyses. Statistical analyses were performed with PASW Statistics 18 software.

## 3. Results

### 3.1. Expression of LARS and DKK4 in Human CRC Tissues

**Figure 1:**
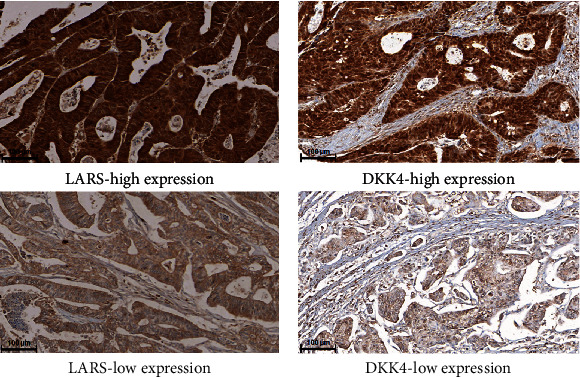
Representative illustrations of immunohistochemical staining for LARS and DKK4 in CRC tissue. (a) Upper: LARS high expression in colorectal cancer tissue. Lower: LARS low expression in colorectal cancer tissue. (b) Upper: DKK4 high expression in colorectal cancer tissue. Lower: DKK4 low expression in colorectal cancer tissue. Magnification 200x for all panels.

The clinical characteristics of the CRC patients enrolled in this study are summarized in Table 1. To examine the expression of LARS in the enrolled CRC patients, immunohistochemistry was performed with an anti-LARS antibody. We observed the high expression of LARS in 468 (72.9%) of the 642 CRC tissue specimens (Table 1). As shown in Figure 1(a), immunostaining was observed in the cytoplasm of the cancer cells. We also examined DKK4 expression in human CRC tissue by immunohistochemistry. A high expression of DKK4 was observed in 494 (76.9%) of the 642 CRC tissue specimens (Figure 1(b) and Table 1).

Until now, there has been no report showing the relationship between LARS expression and DKK4 expression in CRC. Thus, in this study, to investigate their relationship in CRC, the chi-squared test was used. In 85% (420 out of 468) of the patient samples in which LARS was highly expressed, DKK4 was also highly expressed (*p* < 0.001). These data suggest that LARS expression is positively correlated with DKK4 expression.

### 3.2. Association between the Expression of LARS and DKK4 and the Clinicopathological Characteristics

Table 2 summarizes the relationship between LARS expression and the clinicopathological features. The tumor stage was classified according to TNM staging, with 92 patients graded as stage 0 and I, 267 as stage II, and 283 as stage III and IV. LARS expression was not significantly correlated with gender, age at the time of surgery, size, grade, tumor location, tumor invasion, or metastasis (all *p* > 0.05; Table 2). However, LARS expression was significantly correlated with TNM stage, N stage, and LN metastasis (*p* < 0.001, *p* = 0.003, and *p* = 0.001, respectively; Table 2). As shown in Table 2, DKK4 expression was not significantly associated with gender, age at the time of surgery, grade, size, location, tumor invasion, LN metastasis, or metastasis (all *p* > 0.05; Table 2). Interestingly, DKK4 expression was inversely related to the TNM stage and N stage (*p* = 0.048 and *p* = 0.022, respectively; Table 2).

### 3.3. LARS Expression and Clinical Outcomes of CRC Patients

**Figure 2:**
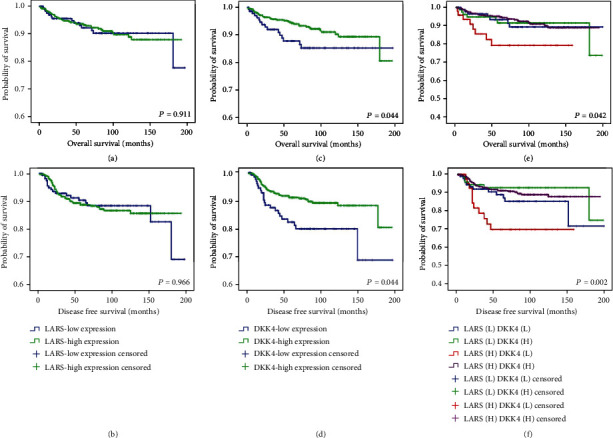
The Kaplan-Meier survival curves in 642 patients with CRC according to the expression levels of LARS and DKK4 and the combination of LARS and DKK4 expression levels. (a) The Kaplan-Meier survival curves for OS in CRC patients. The patients were dichotomized according to LARS expression. (b) The Kaplan-Meier survival curves for DFS in CRC patients. The patients were dichotomized according to LARS expression. (c) The Kaplan-Meier survival curves for OS in CRC patients. The patients were dichotomized according to DKK4 expression. (d) The Kaplan-Meier survival curves for DFS in CRC patients. Patients dichotomized according to DKK4 expression. (e) The Kaplan-Meier survival curves for OS in 642 CRC patients according to the combination of LARS and DKK4 expression levels. (f) The Kaplan-Meier survival curves for DFS in 642 CRC patients according to the combination of LARS and DKK4 expression levels. OS: overall survival; DFS: disease-free survival.

As expected, the OS of CRC patients enrolled in this study was significantly correlated with TNM stage, tumor invasion, N stage, LN metastasis, and metastasis (*p* = 0.005, *p* < 0.001, *p* < 0.001, *p* = 0.014, and *p* < 0.001, respectively; Table 3). In addition, the DFS of CRC patients was significantly correlated with TNM stage, tumor invasion, N stage, LN metastasis, and metastasis (*p* < 0.001, *p* < 0.001, *p* < 0.001, *p* = 0.001, and *p* < 0.001, respectively; Table 4). As we found that LARS expression was related to TNM stage, N stage, and LN metastasis, a log-rank test with the Kaplan-Meier survival curves was used to evaluate whether LARS expression affected the survival of patients with surgically resected CRC. The mean ± SD of the OS for the 642 patients was 180.40 ± 2.65 (95% CI: 175.21–185.59) months. The mean OS for patients with LARS high expression (177.04 ± 2.67 (95% CI: 171.80–182.29) months) was a little bit lower as compared to patients with LARS low expression (180.58 ± 4.71 (95% CI: 171.35–189.81) months) (*p* = 0.911, log-rank test) (Figure 2(a)). The mean ± SD of the DFS for the 642 patients was 174.35 ± 3.04 (95% CI: 168.39–180.31) months. The mean DFS in the LARS high expression group (171.69 ± 2.99 (95% CI: 165.83–177.56) months) was a little bit lower as compared to the LARS low expression group (173.38 ± 5.79 (95% CI: 162.04–184.73) months) (*p* = 0.966, log-rank test) (Figure 2(b)). Unexpectedly, among the 642 analyzed patients, the OS and DFS of the LARS high expression group (*n* = 468) were not significantly different compared to the LARS low expression group (*n* = 174) (*p* = 0.911 and *p* = 0.966; Figures 2(a) and 2(b)).

### 3.4. DKK4 Expression and Clinical Outcomes of CRC Patients

As we observed that DKK4 expression was inversely related to the TNM stage and N stage, we used a log-rank test with the Kaplan-Meier estimates to determine whether DKK4 expression was a useful prognostic factor for the survival of patients with surgically resected CRC. The mean OS for patients with DKK4 high expression (181.51 ± 2.84 (95% CI: 175.94–187.08) months) was significantly higher than in patients with DKK4 low expression (174.31 ± 5.75 (95% CI: 163.04–185.58) months) (*p* = 0.044, log-rank test) (Figure 2(c)). The mean DFS in the DKK4 high expression group (177.81 ± 3.05 (95% CI: 171.83–183.78) months) was significantly higher compared to the DKK4 low expression group (159.00 ± 8.13 (95% CI: 143.07–174.93) months) (*p* = 0.004, log-rank test) (Figure 2(d)). In addition, the Cox regression analysis showed that DKK4 low expression was significantly associated with lower OS and DFS compared to DKK4 high expression (OS: HR = 1.82, 95% CI: 1.01–3.30, *p* = 0.047; DFS: HR = 2.08, 95% CI: 1.25–3.45, *p* = 0.005, respectively; Tables 3 and 4).

We performed multivariate analysis to evaluate the independent prognostic significance of DKK4 expression. We tested TNM stage, tumor invasion, metastasis, N stage, and LN metastasis in the Cox proportional hazard model. In CRC, N stage (stage 2), LN metastasis, and metastasis were independent prognostic factors for OS (*p* = 0.002, *p* = 0.023, and *p* = 0.001, respectively; Table 3). However, DKK4 expression, tumor invasion, and TNM stage were not independent prognostic factors for OS (*p* = 0.081, *p* = 0.063, and *p* = 0.697, respectively; Table 5). DKK4 expression and tumor invasion were independent prognostic factors for DFS (HR = 2.01, 95% CI: 1.20–3.37, *p* = 0.008; HR = 3.02, 95% CI: 1.22–7.46, *p* = 0.017, respectively; Table 4). These data suggest that DKK4 low expression can be used as an independent predictor of relapse in CRC patients.

### 3.5. Prognostic Significance of Combinations of LARS and DKK4 Expressions in CRC Patients

The above observations showed that LARS expression was positively related to the TNM stage, N stage, and nodal involvement, which are known factors influencing the survival of CRC patients. However, LARS expression did not affect the OS and DFS of CRC patients. These data indicate that LARS function was influenced by other proteins in regulating the survival of CRC patients. In contrast, DKK4 expression was inversely related to the TNM stage and N stage and affected the DFS and OS of CRC patients. Thus, we hypothesized that the combination of LARS and DKK4 expressions can be a useful predictor of DFS and OS in CRC patients. To test this hypothesis, we analyzed the OS and DFS using different combinations of LARS and DKK4 expressions. Interestingly, the LARS high/DKK4 low expression group showed significantly lower OS and DFS compared to the LARS high/DKK4 high expression group (*p* = 0.042 and *p* = 0.002, respectively; Figures 2(e) and 2(f)). In addition, the Cox regression analysis showed that the LARS high/DKK4 low expression group was significantly associated with a worse prognosis (OS: HR = 2.92, 95% CI: 1.34–6.40, *p* = 0.007; DFS: HR = 3.21, 95% CI: 1.64–6.29, *p* = 0.001, respectively; Tables 5 and 6). We performed multivariate analysis to evaluate the independent prognostic significance of the combination of LARS and DKK4 expressions. We tested TNM stage, tumor invasion, N stage, LN metastasis, metastasis, and the combination of LARS and DKK4 expressions in the Cox proportional hazard model. For OS in CRC patients, N stage (2), metastasis, and LARS high/DKK4 low expression were independent poor prognostic factors (HR = 3.66, 95% CI: 1.69–7.94, *p* = 0.001; HR = 4.92, 95% CI: 1.95–12.39, *p* = 0.001; and HR = 2.75, 95% CI: 1.21–6.25, *p* = 0.015, respectively; Table 5). For DFS in CRC patients, tumor invasion and LARS high/DKK4 low expression were independent poor prognostic factors (HR = 3.14, 95% CI: 1.27–7.76, *p* = 0.013; HR = 3.04, 95% CI: 1.52–6.09, *p* = 0.002, respectively; Table 6). These data suggest that the combination of DKK4 low expression and LARS high expression significantly reduced the OS and DFS rates of CRC patients. Taken together, it is very useful to analyze the expression of LARS and DKK4 simultaneously when examining tumor sections at the diagnosis of CRC to predict the recurrence and OS of CRC patients.

## 4. Discussion

LARS expression was reported to be upregulated in several cancers including lung cancer [6, 8]. Thus, many investigators have tried to develop novel anticancer agents targeting LARS [7–13]. However, the biological and clinical significance of LARS expression in CRC has not been reported yet. We found that LARS expression was significantly associated with TNM stage, N stage, and LN metastasis. These findings are consistent with a previous report showing that LARS expression was related to the growth and migration of lung cancer cells [6]. Generally, it has been known that the OS and DFS of CRC patients are affected by the TNM stage, N stage, LN metastasis, and metastasis. Unexpectedly, our study showed that the OS and DFS of patients with LARS high expression (468 cases) were not different compared to patients with LARS low expression. These results are similar to a previous study showing that LARS expression was not correlated with the OS of patients with lung cancer, even though LARS expression was associated with mTORC1 activity indicated by the increased expression of p-S6 kinase [8]. These data indicate that the effect of LARS expression on the survival and relapse of CRC patients could be affected by other factors involved in the regulation of TNM stage, N stage, LN metastasis, or metastasis. Further studies to identify the proteins that can affect the function of LARS are needed in the future.

DKK4 is known as an inhibitor of the Wnt/*β*-catenin pathway by binding to LRP5/6 and is induced by *β*-catenin [19, 20, 25]. The Wnt/*β*-catenin signaling pathway is a major pathway in the development of CRC and is activated by the somatic mutations of signaling molecules, such as APC (adenomatous polyposis coli), FBXW7 (F-box and WD repeat domain containing 7), and CTNNB1 (catenin beta1) proteins [36, 37]. Many investigators have examined the role of DKK4 expression in several cancers, but there are still controversies about the role of DKK4 in cancer, depending upon the cancer type [25–30]. In this study, we found that LARS expression was positively related to DKK4 expression. Very interestingly, DKK4 expression showed a significant negative correlation with the TNM stage and N stage, which is a poor prognostic factor for CRC patients. Our present findings are similar to other reports showing that the upregulation of DKK4 by T3 inhibited the invasion and metastasis of hepatoma cells through the degradation of *β*-catenin [32, 38]. Additionally, Fatima et al. [20] demonstrated that DKK4 overexpression inhibited cell proliferation, colony formation, cell migration, and tumor growth by inhibiting *β*-catenin in hepatocellular carcinoma. The negative association of DKK4 with TNM stage and LN metastasis may have been mediated by decreased *β*-catenin due to the inhibitory effect of DKK4, but we did not examine the expression of *β*-catenin in CRC patients. Thus, immunohistochemical staining using an anti-*β*-catenin antibody in the CRC patients enrolled in this study is required. Meanwhile, the OS and DFS of the DKK4 high expression group (494 cases) were significantly higher than those of the DKK4 low expression group, but the Cox regression analysis showed that only N stage (stage 2), LN metastasis, and metastasis were independent poor prognostic factors of OS in CRC patients, and DKK4 expression alone was not an independent prognostic factor of OS. Interestingly, tumor invasion and DKK4 low expression were independent poor prognostic factors of DFS in CRC patients. These data suggest that DKK4 low expression at diagnosis could be used as a predictor of recurrence in CRC patients. The molecular mechanism by which DKK4 high expression predicts a good prognosis for CRC patients and inhibits TNM stage progression and LN metastasis is not clear. RNA sequencing, migration assays, and *in vivo* experiments using DKK4 knockdown or overexpressing CRC cell lines are required to determine the molecular mechanism by which DKK4 expression is negatively correlated with the TNM stage and N stage in CRC. In contrast to our findings, a recent study by Tsukui et al. [30] reported that strong DKK4 expression was related to LN metastasis and a poor prognostic factor of CRC, even though they evaluated a smaller number of CRC patients (*n* = 122) compared to our study (*n* = 642). They showed that DKK4 high expression was associated with somatic gene mutations in the Wnt signaling pathway such as *APC*, *FBXW7*, or *CTNNB1* genes [30]. These discrepancies between our results and those of Tsukui may be explained by the use of different antibodies and the immunohistochemical reaction conditions used to examine the expression of DKK4, the small number of enrolled patients, and the heterogeneous genetic background of CRC. In this study, we did not investigate the somatic gene mutations of the enrolled CRC patients. Further studies investigating genetic mutation of CRC patients with DKK4 expression will be helpful to confirm the potential prognostic factor of DKK4.

We found that patients with LARS high expression and DKK4 low expression had significantly lower OS and DFS than those with LARS high expression and DKK4 high expression. The Cox regression analysis showed that N stage (stage 2), metastasis, and LARS high/DKK4 low expression were independent poor prognostic factors of OS in CRC patients. We also found that tumor invasion and LARS high/DKK4 low expression were independent poor prognostic factors of DFS. Thus, these data suggest that immunostaining for LARS and DKK4 in CRC samples at diagnosis may be useful in predicting the relapse and survival of CRC patients.

To our knowledge, no previous reports have shown the molecular relationship between LARS and DKK4. In the future, studies investigating the molecular network of LARS and DKK4 in human CRC cell lines are needed to clarify the molecular mechanism by which DKK4 low expression in the presence of LARS high expression acts as an independent poor prognostic factor in CRC patients. Our study provided useful findings on the clinical significance of LARS and DKK4 in CRC. However, it also had some limitations. First, the mortality and the recurrence rates during the study were too low, whereas the censored number was large. Second, information on the genetic mutations of CRC patients was not included. Third, the molecular mechanisms by which DKK4 high expression alone and in combination with LARS high expression may be a good prognostic factor in CRC were not clarified in this study. Fourth, we could not exclude the influence of various therapeutic regimen after surgery in the CRC patients in the study results because of the retrospective nature of the study.

## 5. Conclusion

Our present results demonstrated that LARS expression was significantly associated with TNM stage, N stage, and LN metastasis. However, its expression was not correlated with the OS and DFS of CRC patients. DKK4 high expression showed an inverse correlation with the TNM stage and N stage and was a good prognostic factor in CRC patients. In addition, in CRC patients with LARS high expression, the prognosis was significantly worsened by DKK4 low expression. This suggests that the molecular classification of combined LARS and DKK4 expressions in primary CRC may be a useful indicator of LN metastasis and relapse. To establish the efficacy of the combination of LARS and DKK4 as a prognostic factor in CRC patients, validation in a large prospective study and mechanistic studies evaluating the molecular interactions of LARS and DKK4 are warranted.

## Figures and Tables

**Table 1:** Clinical characteristics and LARS and DKK4 expressions in the enrolled CRC patients.

Characteristics	*N*	(%)
Sex		
Men	368	(57.3%)
Women	274	(42.7%)
Age (year)		
<65	356	(55.5%)
≥65	286	(44.5%)
Grade		
Well differentiated	359	(55.9%)
Moderately differentiated	244	(38.0%)
Poorly differentiated and undifferentiated	39	(6.1%)
Size (cm)		
<5	238	(37.1%)
≥5	404	(62.9%)
Location		
Left side (R/S/D)	506	(78.8%)
Right side (T/A/C)	136	(21.2%)
TNM stage		
0+I+II	359	(55.9%)
III+IV	283	(44.1%)
Tumor invasion		
T1-T3	615	(95.8%)
T4	27	(4.2%)
N stage		
0	369	(57.5%)
1	178	(27.7%)
2	95	(14.8%)
LN metastasis		
(-)	369	(57.5%)
(+)	273	(42.5%)
Metastasis		
(-)	604	(94.1%)
(+)	38	(5.9%)
LARS		
Low expression	174	(27.1%)
High expression	468	(72.9%)
DKK4		
Low expression	148	(23.1%)
High expression	494	(76.9%)

Abbreviations: LARS: leucyl-tRNA synthetase; DKK4: Dickkopf 4; LN: lymph node; TNM: tumor-node-metastasis; R: rectum; S: sigmoid colon; D: descending colon; T: transverse colon; A: ascending colon; C: cecum.

**Table 2:** The relationship between clinical characteristics and immunohistochemistry expressions.

Characteristics	LARS expression			DKK4 expression		
	Low	High	*p* value	Low	High	*p* value
	No. of patients (%)	No. of patients (%)		No. of patients (%)	No. of patients (%)	
*Sex*						
Men	101 (27.4)	267 (72.6)	0.858	77 (20.9)	291 (79.1)	0.155
Women	73 (26.6)	201 (73.4)		71(25.9)	203 (74.1)	
*Age*						
<65	93 (26.1)	263 (73.9)	0.533	79 (22.2)	277 (77.8)	0.573
≥65	81 (28.3)	205 (71.7)		69 (24.1)	217 (75.9)	
*Grade*						
Well differentiated	102 (28.4)	257 (71.6)	0.702	78 (21.7)	281 (78.3)	0.219
Moderately differentiated	62 (25.4)	182 (74.6)		64 (26.2)	180 (73.8)	
Poorly differentiated and undifferentiated	10 (25.6)	29 (74.4)		6 (15.4)	33 (84.6)	
*Size (cm)*						
<5	58 (24.4)	180 (75.6)	0.270	46 (19.3)	192(80.7)	0.099
≥5	116 (28.7)	288 (71.3)		102 (25.2)	302 (74.8)	
*Location*						
Left side (R/S/D)	142 (28.1)	364 (71.9)	0.329	122 (24.1)	384 (75.9)	0.252
Right side (T/A/C)	32 (23.5)	104 (76.5)		26 (19.1)	110 (80.9)	
*TNM stage*						
0+I+II	117 (32.6)	242(67.4)	<0.001^∗∗∗^	72 (20.1)	287 (79.9)	0.048^∗^
III+IV	57 (20.1)	226 (79.9)		76 (26.9)	207 (73.1)	
*Tumor invasion*						
T1-T3	169 (27.5)	446 (72.5)	0.381	139 (22.6)	476 (77.4)	0.240
T4	5 (18.5)	22 (81.5)		9 (33.3)	18 (66.7)	
*N stage*						
0	119 (32.2)	250 (67.8)	0.003^∗∗^	75 (20.3)	294 (79.7)	0.022^∗^
1	35 (19.7)	143 (80.3)		41 (23.0)	137 (77.0)	
2	20 (21.1)	75 (78.9)		32 (33.7)	63 (66.3)	
*LN metastasis*						
(-)	119 (32.2)	250 (67.8)	0.001^∗∗∗^	75 (20.3)	294 (79.7)	0.059
(+)	55 (20.1)	218 (79.9)		73 (26.7)	200 (73.3)	
*Metastasis*						
(-)	168 (27.8)	436 (72.2)	0.132	139 (23.0)	465 (77.0)	1.000
(+)	6 (15.8)	32(84.2)		9 (23.7)	29 (76.3)	

Abbreviations: LARS: leucyl-tRNA synthetase; DKK4: Dickkopf 4; LN: lymph node; TNM: tumor-node-metastasis; R: rectum; S: sigmoid colon; D: descending colon; T: transverse colon; A: ascending colon; C: cecum. ^∗^*p* value is based on the chi-squared test. ^∗^*p* < 0.05, ^∗∗^*p* < 0.01, and ^∗∗∗^*p* < 0.001.

**Table 3:** Death hazard ratios of DKK4 expression and clinical characteristics based on OS.

Characteristics	Univariate	Multivariate analysis		
	*p* value	HR	(95% CI)	*p* value
Age (<65 vs. ≥65)	0.291	—	—	—
Gender (male vs. female)	0.148	—	—	—
Grade	0.959	—	—	—
Size	0.054	—	—	—
Location	0.464	—	—	—
DKK 4 expression				
Low vs. high	0.044^∗^	1.72	0.93-3.16	0.081
Tumor invasion				
T4	<0.001^∗∗∗^	2.60	0.95-7.10	0.063
N stage				
2	<0.001^∗∗∗^	3.48	1.61-7.58	0.002^∗∗^
LN metastasis				
(+)	0.014^∗^	4.88	1.24-19.18	0.023^∗^
TNM stage				
III+IV	0.005^∗∗^	1.38	0.28-6.85	0.697
Metastasis				
(+)	<0.001^∗∗∗^	4.79	1.90-12.10	0.001^∗∗^

Abbreviations: DKK4: Dickkopf 4; LN: lymph node; HR: hazard ratio; CI: confidence interval. Univariate analysis was performed by the log-rank test. Multivariate analysis retained only statistically significant (*p* < 0.05) prognostic factors in the Cox regression model. The variables tested were N stage, LN metastasis, TNM stage, metastasis, and DKK4 expression. ^∗^*p* < 0.05, ^∗∗^*p* < 0.01, and ^∗∗∗^*p* < 0.001.

**Table 4:** Death hazard ratios of DKK4 expression and clinical characteristics based on DFS.

Characteristics	Univariate	Multivariate analysis		
	*p* value	HR	(95% CI)	*p* value
Age (<65 vs. ≥65)	0.565	—	—	—
Gender (male vs. female)	0.335	—	—	—
Grade	0.852	—	—	—
Size	0.555	—	—	—
Location	0.343	—	—	—
DKK 4 expression				
Low vs. high	0.004^∗∗^	2.01	1.20-3.37	0.008^∗∗^
Tumor invasion				
T4	<0.001^∗∗∗^	3.02	1.22-7.46	0.017^∗^
N stage				
2	<0.001^∗∗∗^	1.60	0.84-3.07	0.156
LN metastasis				
(+)	0.001^∗∗^	1.15	0.30-4.37	0.840
TNM stage				
III+IV	<0.001^∗∗∗^	3.37	0.85-13.46	0.085
Metastasis				
(+)	<0.001^∗∗∗^	2.35	0.88-6.28	0.088

Abbreviations: DKK4: Dickkopf 4; LN: lymph node; HR: hazard ratio; CI: confidence interval. Univariate analysis was performed by the log-rank test. Multivariate analysis retained only statistically significant (*p* < 0.05) prognostic factors in the Cox regression model. The variables tested were TNM stage, N stage, LN metastasis, metastasis, and DKK4 expression. ^∗^*p* < 0.05, ^∗∗^*p* < 0.01, and ^∗∗∗^*p* < 0.001.

**Table 5:** Death hazard ratio of combined LARS and DKK4 expressions based on OS.

Characteristics	Univariate		*p* value	Multivariate		*p* value
	HR	(95% CI)		HR	(95% CI)	
Expression						
L(L)D(L)	1.19	(0.61, 2.94)	0.462	1.37	(0.62, 3.02)	0.443
L(L)D(H)	1.06	(0.44, 2.57)	0.898	1.49	(0.59, 3.75)	0.397
L(H)D(L)	2.92	(1.34, 6.40)	0.007^∗∗^	2.75	(1.21, 6.25)	0.015^∗^
L(H)D(H)	Ref.			Ref.		
Tumor invasion						
T4	4.96	(2.11, 11.70)	<0.001^∗∗∗^	2.51	(0.92, 6.85)	0.073
LN metastasis						
(+)	1.98	(1.14, 3.45)	0.016^∗^	3.74	(0.81, 17.21)	0.091
N stage						
2	4.02	(2.13, 7.58)	<0.001^∗∗∗^	3.66	(1.69, 7.94)	0.001^∗∗∗^
1	3.61	(1.69, 7.69)	0.001^∗∗^			
TNM stage						
III+IV	2.21	(1.26, 3.87)	0.006^∗∗^	1.54	(0.24, 6.21)	0.799
Metastasis						
(+)	6.09	(3.03, 12.24)	<0.001^∗∗∗^	4.92	(1.95, 12.39)	0.001^∗∗∗^

Abbreviations: LARS: leucyl-tRNA synthetase; DKK4: Dickkopf 4; L(L)D(L): LARS (low) DKK4 (low); L(L)D(H): LARS (low) DKK4 (high); L(H)D(L): LARS (high) DKK4 (low); L(H)D(H): LARS (high) DKK4 (high); OS: overall survival; HR: hazard ratio; CI: confidence interval. Univariate analysis was performed by Cox regression analysis. Multivariate analysis retained only the statistically significant (*p* < 0.05) prognostic factors in the Cox regression model. The variables tested were lymph node metastasis, N stage, TNM stage, and the expression of LARS and DKK4 in combination. ^∗^*p* < 0.05, ^∗∗^*p* < 0.01, and ^∗∗∗^*p* < 0.001.

**Table 6:** Death hazard ratio of combined LARS and DKK4 expressions based on DFS.

Characteristics	Univariate		*p* value	Multivariate		*p* value
	HR	(95% CI)		HR	(95% CI)	
Expression						
L(L)D(L)	1.50	(0.79, 2.88)	0.22	1.59	(0.82, 3.07)	0.168
L(L)D(H)	1.25	(0.52, 2.96)	0.619	1.18	(0.48, 2.87)	0.722
L(H)D(L)	3.21	(1.64, 6.29)	0.001^∗∗^	3.04	(1.52, 6.09)	0.002^∗∗^
L(H)D(H)	Ref.			Ref.		
Tumor invasion						
T4	5.27	(2.39, 11.60)	<0.001^∗∗∗^	3.14	(1.27, 7.76)	0.013^∗^
LN metastasis						
(+)	2.29	(1.41, 3.73)	0.001^∗∗^	1.23	(0.32, 4.69)	0.761
N stage						
2	3.13	(1.69, 5.81)	<0.001^∗∗∗^	1.69	(0.88, 3.25)	0.113
1	1.62	(0.86, 3.08)	0.138			
TNM stage						
III+IV	2.86	(1.73, 4.72)	<0.001^∗∗∗^	3.55	(0.88, 14.26)	0.074
Metastasis						
(+)	5.14	(2.61, 10.12)	<0.001^∗∗∗^	2.36	(0.89, 6.26)	0.086

Abbreviations: LARS: leucyl-tRNA synthetase; DKK4: Dickkopf 4; L(L)D(L), LARS (low) DKK4 (low); L(L)D(H): LARS (low) DKK4 (high); L(H)D(L): LARS (high) DKK4 (low); L(H)D(H): LARS (high) DKK4 (high); DFS: disease-free survival; HR: hazard ratio; CI: confidence interval. Univariate analysis was performed by Cox regression analysis. Multivariate analysis retained only the statistically significant (*p* < 0.05) prognostic factors in the Cox regression model. The variables tested were lymph node metastasis, N stage, TNM stage, metastasis, and the expression of LARS and DKK4 in combination. ^∗^*p* < 0.05, ^∗∗^*p* < 0.01, and ^∗∗∗^*p* < 0.001.

## Data Availability

All data were collected and recorded in Microsoft Excel. The clinical materials are hematoxylin and eosin (H&E) and immunostained slides, which are stored in the Pathology Department of Dong-A University Medical Center. All data generated or analyzed during this study are available from the corresponding author upon reasonable request.

## References

[1] Siegel R. L., Miller K. D., Jemal A. (2019). Cancer statistics, 2019. *CA: a Cancer Journal for Clinicians*.

[2] Goel A., Arnold C. N., Boland C. R. (2001). Multistep progression of colorectal cancer in the setting of microsatellite instability: new details and novel insights. *Gastroenterology*.

[3] Zhou W., Goodman S. N., Galizia G. (2002). Counting alleles to predict recurrence of early-stage colorectal cancers. *Lancet*.

[4] Han J. M., Jeong S. J., Park M. C. (2012). Leucyl-tRNA synthetase is an intracellular leucine sensor for the mTORC1-signaling pathway. *Cell*.

[5] Bonfils G., Jaquenoud M., Bontron S., Ostrowicz C., Ungermann C., De Virgilio C. (2012). Leucyl-tRNA synthetase controls TORC1 via the EGO complex. *Molecular Cell*.

[6] Shin S. H., Kim H. S., Jung S. H., Xu H. D., Jeong Y. B., Chung Y. J. (2008). Implication of leucyl-tRNA synthetase 1 (LARS1) over-expression in growth and migration of lung cancer cells detected by siRNA targeted knock-down analysis. *Experimental Molecular Medicine*.

[7] Gao G., Yao Y., Li K. (2015). A human leucyl-tRNA synthetase as an anticancer target. *Oncotargets and Therapy*.

[8] Kim E. Y., Lee J. G., Lee J. M. (2019). Therapeutic effects of the novel Leucyl-tRNA synthetase inhibitor BC-LI-0186 in non-small cell lung cancer. *Therapeutic Advances in Medical Oncology*.

[9] Yoon S., Kim J. H., Yoon I. (2016). Discovery of (*S*)-4-isobutyloxazolidin-2-one as a novel leucyl-tRNA synthetase (LRS)-targeted mTORC1 inhibitor. *Bioorganic & Medicinal Chemistry Letters*.

[10] Yoon S., Kim J. H., Kim S. E. (2016). Discovery of leucyladenylate sulfamates as novel leucyl-tRNA synthetase (LRS)-targeted mammalian target of rapamycin complex 1 (mTORC1) inhibitors. *Jounal of Medicinal Chemistery*.

[11] Yoon S., Kim J. H., Koh Y. (2017). Discovery of simplified leucyladenylate sulfamates as novel leucyl-tRNA synthetase (LRS)-targeted mammalian target of rapamycin complex 1 (mTORC1) inhibitors. *Bioorganic & Medicinal Chemistry Letters*.

[12] Yoon S., Zuo D., Kim J. H. (2018). Discovery of novel leucyladenylate sulfamate surrogates as leucyl-tRNA synthetase (LRS)-targeted mammalian target of rapamycin complex 1 (mTORC1) inhibitors. *Bioorganic & Medicinal Chemistry Letters*.

[13] Yoon S., Kim S. E., Kim J. H. (2019). Structure-activity relationship of leucyladenylate sulfamate analogues as leucyl-tRNA synthetase (LRS)-targeting inhibitors of mammalian target of rapamycin complex 1 (mTORC1). *Bioorganic & Medicinal Chemistry Letters*.

[14] Fatima S., Lee N. P., Luk J. M. (2011). Dickkopfs and Wnt/*β*-catenin signalling in liver cancer. *World Journal of Clinical Oncology*.

[15] Niehrs C. (2006). Function and biological roles of the Dickkopf family of Wnt modulators. *Oncogene*.

[16] Rosenbluh J., Wang X., Hahn W. C. (2014). Genomic insights into WNT/*β*-catenin signaling. *Trends in Pharmacological Sciences*.

[17] Logan C. Y., Nusse R. (2004). The Wnt signaling pathway in development and disease. *Annual Review of Cell and Developmental Biology*.

[18] Fang D., Hawke D., Zheng Y. (2007). Phosphorylation of *β*-catenin by AKT promotes *β*-catenin transcriptional activity. *The Journal of Biological Chemistry*.

[19] Hirata H., Hinoda Y., Majid S. (2011). DICKKOPF-4 activates the noncanonical c-Jun-NH_2_ kinase signaling pathway while inhibiting the Wnt-canonical pathway in human renal cell carcinoma. *Cancer*.

[20] Fatima S., Lee N. P., Tsang F. H. (2012). Dickkopf 4 (DKK4) acts on Wnt/*β*-catenin pathway by influencing *β*-catenin in hepatocellular carcinoma. *Oncogene*.

[21] Hu Y., Liu M., Xu S. (2020). The clinical significance of Dickkopf Wnt signaling pathway inhibitor gene family in head and neck squamous cell carcinoma. *Medical Science Monitor*.

[22] Katase N., Nagano K., Fujita S. (2020). DKK3 expression and function in head and neck squamous cell carcinoma and other cancers. *Journal of Oral Biosciences*.

[23] Gao Y. B., Chen Z. L., Li J. G. (2014). Genetic landscape of esophageal squamous cell carcinoma. *Nature Genetics*.

[24] Wang S., Wei H., Zhang S. (2017). Dickkopf-4 is frequently overexpressed in epithelial ovarian carcinoma and promotes tumor invasion. *BMC Cancer*.

[25] Matsui A., Yamaguchi T., Maekawa S. (2009). DICKKOPF-4 and -2 genes are upregulated in human colorectal cancer. *Cancer Sciences*.

[26] Pendás-Franco N., Aguilera O., Pereira F., González-Sancho J. M., Muñoz A. (2008). Vitamin D and Wnt/*β*-catenin pathway in colon cancer: role and regulation of DICKKOPF genes. *Anticancer Research*.

[27] Pendás-Franco N., García J. M., Peña C. (2008). DICKKOPF-4 is induced by TCF/beta-catenin and upregulated in human colon cancer, promotes tumour cell invasion and angiogenesis and is repressed by 1alpha,25-dihydroxyvitamin D3. *Oncogene*.

[28] Ebert M. P., Tänzer M., Balluff B. (2012). TFAP2E-DKK4 and chemoresistance in colorectal cancer. *The New England Journal of Medicine*.

[29] He S., Shen J., Hu N., Xu X., Li J. (2017). DKK4 enhances resistance to chemotherapeutics 5-Fu and YN968D1 in colorectal cancer cells. *Oncology Letters*.

[30] Tsukui Y., Yamaguchi T., Maekawa S., Takano S., Sato T., Enomoto N. (2019). Dickkopf-4 gene expression is associated with differentiation and lymph node metastasis in colorectal cancer. *Journal of Gastroenterology and Hepatology Open*.

[31] Baehs S., Herbst A., Thieme S. E. (2009). Dickkopf-4 is frequently down-regulated and inhibits growth of colorectal cancer cells. *Cancer Letters*.

[32] Liao C. H., Yeh C. T., Huang Y. H. (2012). Dickkopf 4 positively regulated by the thyroid hormone receptor suppresses cell invasion in human hepatoma cells. *Hepatology*.

[33] Lan Y. T., Yang S. H., Chang S. C. (2012). Analysis of the seventh edition of American Joint Committee on colon cancer staging. *International Journal of Colorectal Disease*.

[34] Hsu F. D., Nielsen T. O., Alkushi A. (2002). Tissue microarrays are an effective quality assurance tool for diagnostic immunohistochemistry. *Modern Pathology*.

[35] Yun S. H., Park M. G., Kim Y. M., Roh M. S., Park J. I. (2017). Expression of chicken ovalbumin upstream promoter-transcription factor II and liver X receptor as prognostic indicators for human colorectal cancer. *Oncology Letters*.

[36] Jiang J. X., Sun C. Y., Tian S., Yu C., Chen M. Y., Zhang H. (2016). Tumor suppressor Fbxw7 antagonizes WNT signaling by targeting *β*-catenin for degradation in pancreatic cancer. *Tumour Biology*.

[37] The Cancer Genome Atlas Network (2012). Comprehensive molecular characterization of human colon and rectal cancer. *Nature*.

[38] Chi H. C., Liao C. H., Huang Y. H. (2013). Thyroid hormone receptor inhibits hepatoma cell migration through transcriptional activation of Dickkopf 4. *Biochemical and Biophysics Research Communications*.

